# The efficacy of anti‐programmed cell death protein 1 therapy among patients with metastatic acral and metastatic mucosal melanoma

**DOI:** 10.1002/cam4.3781

**Published:** 2021-03-08

**Authors:** Dai Ogata, Lauren E. Haydu, Isabella C. Glitza, Sapna P. Patel, Hussein A. Tawbi, Jennifer L. McQuade, Adi Diab, Suhendan Ekmekcioglu, Michael K. Wong, Michael A. Davies, Rodabe N. Amaria

**Affiliations:** ^1^ Department of Melanoma Medical Oncology MD Anderson Cancer Center Houston TX USA; ^2^ Department of Dermatologic Oncology National Cancer Center Hospital Tokyo Japan; ^3^ Department of Surgical Oncology MD Anderson Cancer Center Houston TX USA

**Keywords:** acral melanoma, anti‐PD‐1, malignant melanoma, mucosal melanoma

## Abstract

**Background:**

Anti‐programmed cell death protein 1 (PD‐1) antibodies are a standard treatment for metastatic melanoma patients. However, the understanding of the efficacy of anti‐PD‐1 for acral melanoma (AM) and mucosal melanoma (MM) is limited as these subtypes are relatively rare compared to cutaneous melanoma (CM).

**Methods:**

This single institution, retrospective cohort study included patients with advanced AM and MM who underwent anti‐PD‐1 therapy for metastatic melanoma between 2012 and 2018. Objective responses were determined using the investigator‐assessed Response Evaluation Criteria in Solid Tumors version 1.1. Progression‐free survival (PFS) and overall survival (OS) were assessed using the Kaplan–Meier method. A Cox regression analysis was performed to identify the factors associated with survival outcomes.

**Results:**

Ninety‐seven patients were identified, 38 (39%) with AM and 59 (61%) with MM. The objective response rates (ORRs) were 21.0% and 15.2% in patients with AM and MM, respectively. The median PFS and OS were 3.6 and 25.7 months for AM patients, and 3.0 and 20.1 months for MM patients, respectively. Elevated serum lactate dehydrogenase (LDH) (AM: hazard ratio [HR], 0.22; 95% confidence interval [CI], 0.06–0.87; *p* = 0.03, MM: HR, 0.20; 95% CI, 0.08–0.53; *p* = 0.001) was significantly associated with shorter OS for both subtypes.

**Conclusions:**

The ORR, PFS, and OS with anti‐PD‐1 therapy were poor in patients with AM and MM compared to those previously reported clinical trials for nonacral CM. High serum LDH was associated with significantly shorter OS.

## 1 INTRODUCTION

Cutaneous melanoma (CM) is the most common form of melanoma that arises from melanocytes in the basal layer of the epidermis of the skin. Melanocytes also develop within the mucosal surfaces of the body and can give rise to mucosal melanoma (MM). Melanocytes in glabrous skin, including the palms of the hands and soles of the feet, can become acral melanoma (AM). ^1^, ^2^, ^3^, ^4^ In Caucasian populations, the primary sites of melanoma include cutaneous (82%), uveal (8%), acral (3%), and mucosal (2%); the remaining 5% are diagnosed as metastases from unknown primary lesions.^5^ AM and MM have distinct genetic and clinical characteristics,^6^, ^7^ a lower somatic mutational burden,^8^, ^9^ and poor prognosis compared to stage‐matched CM.^10^, ^11^ Most patients with metastatic AM and MM are treated with immune checkpoint inhibitor therapy due to the low prevalence of targetable mutations in these tumor types.^12^, ^13^

Antibodies against programmed cell death receptor 1 (PD‐1) constitute a standard therapy for the management of patients with metastatic melanoma of all subtypes. Recently, the data regarding activity of anti‐PD1 treatment in AM and MM has grown significantly. Shoushtari et al. first described an objective response rate (ORR) of 32% in patients with AM (n = 25) and 23% in those with MM (n = 35).^14^ More recently, it has been reported across a series of predominantly retrospective studies that the ORR achieved with anti‐PD‐1 was 14.0–16.6% and the median overall survival (OS) was 18.2–25.8 months in AM patients, and 0–23.2% and 11.5–20.2 months for MM patients, respectively.^15^, ^16^, ^17^, ^18^, ^19^, ^20^ In contrast, in the Checkmate067 trial the ORR for nivolumab was 43.7% for all melanoma subtypes, with median PFS 6.9 months and median OS 36.9 months for CM patients.^21^ Data thus far suggest that the AM and MM subtypes do not respond as robustly to anti‐PD‐1 therapy as CM. However, the efficacy of anti‐PD‐1 blockade may vary outside of the clinical trial setting, or in different ethnic populations.

Thus, we studied the patient characteristics and survival outcomes of a retrospective cohort of patients from a single U.S. institution diagnosed with AM or MM who received treatment with FDA‐approved single‐agent anti‐PD‐1 (nivolumab or pembrolizumab) therapy as the standard of care for metastatic or unresectable disease.

## 2 MATERIALS AND METHODS

### 2.1 Patients and methods

Under an Institutional Review Board‐approved protocol, patients at the MD Anderson Cancer Center with AM and MM who received at least one dose of single‐agent nivolumab or pembrolizumab between 2012 and 2018 for metastatic or unresectable disease melanoma were identified. Clinical information was retrieved from electronic medical records, including sex; age; ethnicity; disease stage; Eastern Cooperative Oncology Group performance status (PS); primary site; sites of metastatic disease at the initiation of anti‐PD1 therapy; the presence of *BRAF*, neuroblastoma rat sarcoma viral oncogene homolog (*NRAS*), or proto‐oncogene receptor tyrosine kinase (*KIT*) mutations; the number and characteristics of prior and subsequent systemic therapies; treatment‐related variables (anti‐PD‐1 agent used, duration of treatment, reasons for discontinuation, and toxicities); and survival status.

### 2.2 Statistical analyses

Objective response to therapy was determined using the investigator‐assessed Response Evaluation Criteria in Solid Tumors version 1.1.^22^ In this study, we restricted the cohort to those patients who had radiologically measurable disease. The ORR was defined as the proportion of patients who achieved a complete response (CR) or a partial response (PR) at any time after the start of treatment. Patients who received one or more doses of therapy without subsequent radiographic evaluation were considered “not evaluable.”

PFS and OS were assessed using the Kaplan–Meier method. PFS was assessed from the date of anti‐PD‐1 treatment initiation to the date of radiologic progression, change in therapy, death, or last follow‐up. The OS was assessed from the date of treatment initiation to the date of death or last follow‐up. Patients who were alive at the last follow‐up were censored. The log‐rank test was used to compare categorical variables. A Cox regression analysis was performed to identify factors associated with outcomes. All statistical analyses were performed using EZR (Saitama Medical Centre, Jichi Medical University, Saitama, Japan), which is a graphical user interface for R (The R Foundation for Statistical Computing, Vienna, Austria).^23^

## 3 RESULTS

### 3.1 Baseline patient characteristics

The demographic characteristics of the study cohort are shown in Table 1. In total, 97 patients (AM, n = 38; MM, n = 59) who underwent treatment with anti‐PD‐1 agents were identified. The median age at treatment initiation was 67 years (range, 19–89), and 54% were women. Caucasian patients (n = 68) represented 70% of the cohort. The PS was rated 0 in 59 patients (61%) and ≥1 in 38 patients (39%). Among patients with AM, 87% of primary tumors arose from the palm or sole and 13% from the nailbed. Among patients with MM, 29% had anorectal, 29% had vulvovaginal, and 40% had head and neck as the primary tumor sites. Seventy‐seven patients (79%) had undergone prior systemic treatments, including 48 patients (50%) who were previously treated with ipilimumab. Seventy‐five patients received pembrolizumab (AM, n = 33; MM, n = 42) and 22 received nivolumab (AM, n = 5; MM, n = 17). Seventy‐three patients (75%) had metastatic disease at the time of PD‐1 treatment; central nervous system (CNS) involvement was present in 15 (15%), and liver involvement was present in 31 (32%). Sixty‐three patients (65%) had one or two metastatic sites and 34 (35%) had three or more metastatic sites. Serum lactate dehydrogenase (LDH) level was above reference values in 18 patients (19%). An alteration in *BRAF*, *KIT*, or *NRAS* was identified in 14% (5/37), 10% (4/37), and 10% (4/37) of the patients with AM and 3% (2/55), 19% (11/55), and 10% (6/55) of the patients with MM, respectively.

**TABLE 1:** Patient characteristics at base line

Variable	No. of patients (%)		
	Total	Acral	Mucosal
Total no. of patients	97	38 (39)	59 (61)
Age at PD‐1 treatment:			
Median [range], y	67 [19–89]	65 [19–87]	69 [35–89]
Sex			
Male	45 (46)	24 (63)	21 (36)
Female	52 (54)	14 (37)	38 (64)
Ethnicity			
Caucasian	68 (70)	22 (58)	46 (78)
Black	5 (5)	2 (5)	3 (5)
Hispanic	16 (17)	9 (24)	7 (12)
Asian	3 (3)	2 (5)	1 (2)
ne	5 (5)	3 (8)	2 (3)
ECOG PS at treatment initiation			
0	59 (61)	21 (55)	38 (64)
≧1	38 (39)	17 (45)	21 (36)
Site			‐
Sole/palm	33 (34)	33 (87)	‐
Nailbed	5 (5)	5 (13)	
Anorectal	17 (17.5)	‐	17 (29)
Vulvovaginal	17 (17.5)	‐	17 (29)
Head/neck	24 (25)	‐	24 (40)
Esophagus	1 (1)	‐	1 (2)
Prior systemic therapy			
Yes	77 (79)	29 (76)	48 (81)
No	20 (21)	9 (24)	11 (19)
Prior immunotherapy (ipilimumab)			
Yes	48 (50)	20 (53)	28 (47)
No	49 (50)	18 (47)	31 (53)
Agent			
Pembrolizumab	75 (77)	33 (87)	42 (71)
Nivolumab	22 (23)	5 (13)	17 (29)
Stage at treatment			
III		15 (40)	Localized: 6 (10)
IV, M1a		3 (8)	Regional and distant: 53 (90)
IV, M1b		7 (18)	
IV, M1c		6 (16)	
IV, M1d		7 (18)	
Brain metastases			
Yes	15 (15)	7 (18)	8 (14)
No	82 (85)	31 (82)	51 (86)
Liver metastases			
Yes	31 (32)	7 (18)	24 (41)
No	66 (68)	31 (82)	35 (59)
LDH level			
>ULN	18 (19)	8 (21)	10 (17)
<ULN	78 (80)	30 (79)	48 (81)
ne	1 (1)	‐	1 (2)
Number of metastases			
1–2	63 (65)	30 (79)	33 (56)
≧3	34 (35)	8 (21)	26 (44)
Mutations			
BRAF V600	7 (7)	5 (14)	2 (3)
NRAS	15 (16)	4 (10)	11 (19)
KIT	10 (10)	4 (10)	6 (10)
Wild‐type	60 (62)	24 (63)	36 (61)
ne	5 (5)	1 (3)	4 (7)

* All of the following variables in the table were data points acquired at treatment initiation: ECOG performance status (PS), stage, Brain metastases, Liver metastases, LDH level, number of metastasis; ne: not evaluated; ULN: upper limit of normal.

### 3.2 Treatment outcomes in patients with AM

Treatment with anti‐PD1 in AM patients achieved an ORR of 21.0% (5.3% CR, 15.8% PR) and a disease control rate (DCR) of 52.6% (Table 2). Progressive disease (PD) was the best response for 47.3% of the patients with AM. In the univariate analysis of clinical factors associated with ORR in patients with AM, we observed no significant associations with *BRAF* mutation status, brain metastasis, sex, LDH level, liver metastasis, ethnicity, prior immunotherapy, or number of metastases (Supplementary Table S1).

**TABLE 2:** Overall response and disease control rate

Outcome	No. of patients (%)	
	Acral (n = 38)	Mucosal (n = 59)
Best response		
CR	2	3
PR	6	6
SD	12	12
PD	18	34
ne	‐	4
ORR	21.0%	15.2%
DCR	54.1%	38.2%

Abbreviations: DCR, disease control rate; ne, not evaluated; OOR, Objective response rate.

**FIGURE 1:**
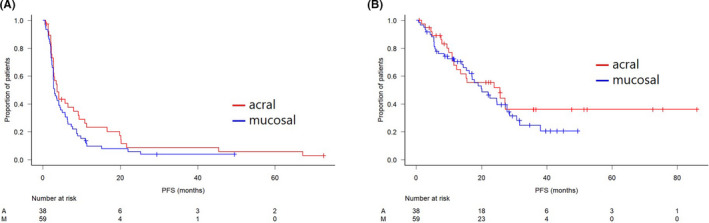
PFS and OS of patients with acral and mucosal melanoma treated with anti‐PD‐1 antibody. **(**A) progression‐free survival; (B) overall survival

With a median follow‐up of 15.6 months, patients with AM had a median PFS of 3.6 months (Figure 1A). The median OS was 25.7 months, and 20 of 39 patients died during the study period (Figure 1B). On multivariate analysis, elevated serum LDH level (hazard ratio [HR], 0.22; 95% confidence interval [CI], 0.06–0.87; *p* = 0.03) was associated with shorter OS. No significant associations were observed for OS with gender, ethnicity, *BRAF* mutation status, prior immune therapy, CNS involvement, liver involvement, or number of metastases (Table 3).

**TABLE 3:** Multivariate analysis of prognostic factors for survival in acral melanoma

Factor	Hazard ratio	*p*‐value
Gender	1.12 (0.33–3.77)	0.86
Ethnicity	0.65 (0.16–2.70)	0.55
BRAF status	0.41 (0.06–2.85)	0.37
Prior immunotherapy	1.25 (0.36–4.34)	0.72
CNS involvement	0.94 (0.18–4.90)	0.94
Liver involvement	0.20 (0.01–3.33)	0.26
Number of metastasis	4.67 (0.47–46.02)	0.19
LDH level	0.22 (0.06–0.87)	0.031

Abbreviation: CNS, central nervous system.

### 3.3 Treatment outcomes in patients with MM

Treatment with anti‐PD1 in patients with MM achieved an ORR of 15.2% (5.1% CR, 10.1% PR) and a DCR of 35.6% (Table 2). PD was the best response in 57.6%. No factors were significantly associated with ORR on univariate analysis (Supplementary Table S2).

With a median follow‐up of 16.5 months, patients with MM had a median PFS of 3.0 months (Figure 1A). The median OS in patients was 20.1 months; 37 of 59 patients died (Figure 1B). In the multivariate analysis, there were significant differences regarding the distribution of elevated serum LDH level (HR, 0.20; 95% CI, 0.08–0.53; *p* = 0.001). However, no significant associations were observed between OS and gender, ethnicity, prior immune therapy, CNS involvement, liver involvement, or more than three organs of metastases (Table 4).

**TABLE 4:** Multivariate analysis of prognostic factors for survival in mucosal melanoma

Factor	Hazard ratio	*p*‐value
Gender	0.95 (0.43–2.12)	0.9
Ethnicity	0.82 (0.30–2.26)	0.7
Prior immunotherapy	1.24 (0.57–2.69)	0.59
CNS involvement	2.88 (0.86–9.56)	0.085
Liver involvement	0.62 (0.28–1.39)	0.25
Number of metastasis	1.63 (0.70–3.79)	0.25
LDH level	0.20 (0.08–0.53)	0.0011

Abbreviation: CNS, central nervous system.

### 3.4 Post‐progression therapy

After treatment discontinuation due to disease progression, 67 patients (69%) received postprogression therapy. Immunotherapy was the most common treatment (n = 32, 33%), followed by cytotoxic chemotherapy (n = 15, 15%) and targeted therapy (n = 14, 14%). Only three patients received ipilimumab and nivolumab combination therapy (Table 5).

**TABLE 5:** Postprogression therapy

	No. of patients (%)		
	Acral (n = 38)	Mucosal (n = 59)	Total (n = 97)
None	14 (37)	16 (27)	30 (31)
Immunotherapy	11 (29)	21 (35)	32 (33)
ipilimumab	4	4	8
nivolumab	3	1	4
pembrolizumab	0	11	11
(±abraxane)			
ipi+nivo	1	2	3
Other	3	3	6
Chemotherapy	3 (8)	12 (20)	15 (15)
Targeted therapy	6 (16)	8 (14)	14 (14)
Radiation	1 (3)	2 (3)	3 (3)
Oncolytic virus	2 (5)	0	2 (2)
Surgery	1 (3)	0	1 (1)

## 4 DISCUSSION

Currently, the evidence on the efficacy of anti‐PD‐1 therapy in patients with metastatic or unresectable AM or MM has grown significantly. Although the response rate in patients with AM (21.0%) and MM (15.2%) was observed in this study to be relatively low compared to previously reported data in patients with CM,^24^, ^25^, ^26^ our results are consistent with previous reports that investigated AM and MM, with reported ORR 14–32% for AM and 0–23% for MM.^16^, ^17^, ^18^, ^19^, ^20^ From these findings, we could consider that anti‐PD‐1 and anti‐CTLA‐4 combination therapy should be the first choice to improve prognosis of AM and MM. However, the efficacy of combination therapy has been shown to be inferior to that of CM and statistically significant difference in the prognostic effect compared to monotherapy was not seen.^17^

Recently, it was reported that anti‐PD‐1 antibodies have limited survival benefit among patients with AM (ORR: 16.6%, median OS: 18.2 months) in Japanese patients.^15^ The authors of that study hypothesized that the difference in the efficacy of anti‐PD‐1 antibody therapy may be due to ethnic differences. It was also reported in a prospective phase II study of toripalimab in China that ORRs and mOS were 14.0% and 16.9 months in AM (n = 50), 0% and 10.3 months in MM (n = 22).^20^ On the contrary, Nathan et al. reported mOS were 25.8 months in AM (n = 55) and 11.5 months in MM (n = 63) in a Caucasian population.^18^ These data suggest that melanomas from patients of East Asian descent may have inferior outcomes with anti‐PD1. In this study, we observed no significant differences for OS between Caucasian and non‐Caucasian patients with AM [HR: 0.65 (0.16–2.70), *p* = 0.55] or MM [HR: 0.82 (0.30–2.26), *p* = 0.7]. However, as this study was an interracial comparison within the United States, and included very few patients of Asian ancestry, we cannot determine if the efficacy of anti‐PD‐1 differs by ethnicity. Future studies should further explore outcomes by ethnicity with immune check point inhibitors, particularly for subtypes that are enriched in specific ethnic subgroups. Although AM and MM fundamentally differ from nonacral CM in their pathogenesis and therapeutic targets,^27^ further studies should include investigation of mechanisms other than mutational burden that can be influenced extrinsically, including factors such as obesity and differential composition of the gut microbiome, for their potential contribution to differential immune responses and efficacy of anti‐PD‐1 in these rare melanoma subtypes.

In the present study, we did not identify a factor significantly associated with ORR in either AM or MM patients. We did observe that elevated serum LDH level was prognostic in our cohort of AM and MM patients treated with anti‐PD‐1. Though AM patients constituted a relatively small proportion and MM patients were not included in the eighth edition of the American Joint Committee on Cancer manual, ^28^ our data support that the LDH level is likely to be a prognostic factor in these melanoma subtypes, consistent with its well‐established prognostic role in CM.^29^

Limitations of this study include its retrospective nature and the small sample size. Although we included both treatment‐naive and previously treated subjects in our study, we observed a lower efficacy of anti‐PD‐1 antibodies in AM and MM patients, and no significant difference based on prior treatment. Our results suggest that because treatment with both ipilimumab and nivolumab is often used for metastatic melanoma, analysis of the outcomes of these rare subtypes using combination therapy should be addressed in the future along with the development of novel immunotherapy strategies.

In conclusion, our cohort of patients with AM and MM treated with anti‐PD‐1 demonstrated worse ORR and OS compared to reported data for nonacral CM patients. Thus, the efficacy of anti‐PD‐1 differs by disease subtype, and perhaps by ethnicity. Moreover, elevated serum LDH level was associated with shorter OS in both AM and MM patients. Therefore, there is a need to develop new targeted or combination therapies for patients with AM and MM that could enhance the efficacy of immune checkpoint inhibitors.

## CONFLICTS OF INTEREST

The authors have no conflicts of interest to declare.

## AUTHOR CONTRIBUTIONS

Study concept and design: Dai Ogata, Suhendan Ekmekcioglu, Lauren E. Haydu, Michael A. Davies and Rodabe N. Amaria; provision of study materials or patients: Isabella C. Glitza, Sapna P. Patel, Hussein A. Tawbi, Jennifer L. McQuade, Adi Diab, Michael K. Wong, Michael A. Davies and Rodabe N. Amaria; collection and assembly of data: Lauren E. Haydu; data analysis and interpretation: Dai Ogata; writing–original draft: Dai Ogata; writing–review and editing: Dai Ogata, Lauren E. Haydu, Michael A. Davies and Rodabe N. Amaria.

## Supporting information

Table S1‐S2

## Data Availability

The data that support the findings of this study are available from the corresponding author, RA, upon reasonable request.
