# Supplementary material for: The efficacy of anti‐programmed cell death protein 1 therapy among patients with metastatic acral and metastatic mucosal melanoma
Source: Cancer Med. 2021 Mar 8;10(7):2293–9. doi: 10.1002/cam4.3781 (PMC7982611; doi:10.1002/cam4.3781)
Supplement: Supplementary file 1 — Table S1‐S2 [file CAM4-10-2293-s001.docx]

Supplementary Table 1. Univariate analysis of best response in acral melanoma

|  | No. of patients (%) | |  |
| --- | --- | --- | --- |
| Variable* | Response (n=8) | No response (n=30) | p-value |
| BRAF status  Mutant  Wild | 0  8 | 4  24 | 0.56 |
| Brain metastasis  Exist  None | 1  7 | 6  24 | 1 |
| Sex  Male  Female | 5  3 | 19  11 | 1 |
| LDH level  <ULN  >ULN | 7  1 | 23  7 | 0.66 |
| Liver metastasis  Exist  none | 2  6 | 5  25 | 0.624 |
| Ethnicity  Caucasian  Non-caucasian  unknown | 6  1  1 | 16  12  2 | 0.298 |
| Immune therapy  Naïve  treated | 4  4 | 14  16 | 1 |
| Number of metastases  ≦2  　≧3 | 7  1 | 23  7 | 0.66 |

*all of the following variables in the table were data points acquired at treatment initiation: Brain metastases, Liver metastases, LDH level, number of metastasis; ULN: upper limit of normal.

Supplementary Table 2. Univariate analysis of best response in mucosal melanoma

|  | No. of patients (%)* | |  |
| --- | --- | --- | --- |
| Variable** | Response (n=9) | No response (n=46) | p-value |
| BRAF status  Mutant  Wild  ne | 0  8  1 | 2  41  3 | 1 |
| Brain metastasis  Exist  None | 0  9 | 8  38 | 0.327 |
| Sex  Male  Female | 4  5 | 15  31 | 0.703 |
| LDH level  <ULN  >ULN | 9  0 | 36  10 | 0.186 |
| Liver metastasis  Exist  none | 2  7 | 18  28 | 0.462 |
| Ethnicity  Caucasian  Non-caucasian  unknown | 6  2  1 | 36  9  1 | 0.333 |
| Immune therapy  Naïve  treated | 5  4 | 25  21 | 1 |
| Number of metastases  ≦2  　≧3 | 6  3 | 25  21 | 0.716 |

*Four cases were excluded because their response could not be evaluated

**all of the following variables in the table were data points acquired at treatment initiation: Brain metastases, Liver metastases, LDH level, number of metastasis; ULN: upper limit of normal.
